# Medical maladies in eating disorders—there is still much work to be done

**DOI:** 10.1186/s40337-023-00790-3

**Published:** 2023-06-05

**Authors:** Allison Nitsch, Phil Mehler

**Affiliations:** 1 ACUTE Center for Eating Disorders at Denver Health, 777 Bannock Street, Denver, CO 80204 USA; 2 Department of Medicine, University of Colorado School of Medicine, 13001 E 17th Pl, Aurora, CO 80045 USA; 3 Eating Recovery Center, 7351 E Lowry Blvd, Denver, CO 80230 USA

**Keywords:** Eating disorders, Medical complications

## Abstract

During the COVID-19 pandemic, the eating disorder clinician community saw an increase in severity and number of people seeking care for eating disorders. Due to this, its even more important for those who work with people with eating disorders to understand the medical complications these people are at risk for. The special issue of the Journal of Eating Disorders provides a comprehensive look at medical complications and also makes apparent deficits in the scientific literature.

## Main text

Anecdotally, since March 2020, we have seen patients presenting to our medical stabilization program for eating disorders at even lower body weights and with more medical complications than in the two decades preceding the COVID-19 pandemic. The literature now supports what was already painfully obvious to those on the frontlines of clinical care for people with eating disorders, that need for care for eating disorders increased and the patients came in sicker during this time period [1]. Many explanations, including isolation, social media, and decreased access to care have been offered. Regardless of the reason, which is likely multifactorial, this makes the focus on medical complications of eating disorders, and thus, this special issue, more critically important. Our goal with this special issue is to provide an up-to-date account of a broad swath of medical complications and their management in people with eating disorders and to highlight knowledge gaps in the field. We hope these articles can also serve as quick reference for primary care providers, general practitioners, and emergency department providers who are unlikely to receive extensive education about the medical care of people with eating disorders during their residency training.

This issue highlights medical complications and management of three growing special patient populations with eating disorders: people with atypical anorexia nervosa, children and adolescents, and transgender individuals [2–4]. Importantly, a review of common presentations of patients with eating disorders to the emergency department is also included [5]. Many people with eating disorders utilize the emergency room as their de facto primary care site and their eating disorder may go undetected by emergency providers [6], or the severity of their disease is underappreciated due to rapid treatment and discharge home when they in fact meet criteria for admission. Both represent missed opportunities for timely intervention.

Disorders of gut-brain interaction, previously referred to as “functional” gastrointestinal disorders, including irritable bowel syndrome, are increasingly recognized as common maladies of people with eating disorders. Thus, we are pleased to include a thorough review of DGBI and their management in the context of eating disorders [7]. Furthermore, a review of the microbiome and its potential burgeoning implications in people with eating disorders is presented [8]. Also presented is a review of pulmonary complications in eating disorders [9]. The lungs have heretofore been considered “spared” by the ravages of eating disorders for many years, however, we believe the review of pulmonary complications, included in this issue, argues to the contrary and shed light on an area which requires further research focus.

Additionally, no review of medical complications of eating disorders would be complete without discussion of cardiovascular, endocrine, renal, and electrolyte complications [10–12]. While in recent years, research has suggested that arrhythmogenic cardiovascular complications are less likely to be the harbinger of death for people with eating disorders they once were thought to be [13], this remains an important area of focus and a need for familiarity by the medical provider treating people with eating disorders. The endocrine system remains a big target for the adverse effects of anorexia nervosa. Lastly, the consequences of eating disorders on the kidneys, including electrolyte disturbances and the potential for inherent chronic kidney injury, are reviewed.

This special medical issue also contains two original research articles, one pertaining to bone health [14] and the other to cause of death in severe anorexia nervosa [15]. The systematic review on bone health in eating disorders highlights the oft irreversible diminution of bone mineral density in anorexia nervosa. The review on cause of death in anorexia nervosa intriguingly found, while higher than the general population, suicide was infrequent while anorexia nervosa and/or medical illness was listed as a cause of death in the majority patients that had been hospitalized for medical stabilization for severe anorexia nervosa and then continued on to residential treatment [15]. This reinforces how deadly eating disorders can be even in the face of access to treatment.

We would like to call upon the eating disorder professional community to further study and publish on medical complications found in avoidant/restrictive food intake disorder, binge eating disorder, and atypical anorexia nervosa. Additionally, studies of these underrepresented eating disorders in the literature as well as future studies of anorexia nervosa and bulimia nervosa should strive to enroll racially, ethnically, gender, and socio-economically diverse participants. Furthermore, we ask internal, family or general practice, and emergency medicine graduate training programs to better educate their residents on screening for eating disorders and the recognition of medical complications of eating disorders to help ensure people with eating disorders are appropriately identified and referred for comprehensive, multidisciplinary care to achieve a sustained recover from their illness.

## Conclusions

Understanding the medical complications of eating disorders is more pertinent than ever given the increase in severity and occurrence of eating disorders in recent years. Further exploration of medical complications of eating disorders, particularly binge eating disorder, “atypical” anorexia nervosa, and avoidant/restrictive food intake disorder, is needed in the scientific literature to better understand medical risks to people with eating disorders and how to manage them.

## Data Availability

Not applicable.
